# Surgery for gastrointestinal metastases of malignant melanoma — a retrospective exploratory study

**DOI:** 10.1186/s12957-019-1663-z

**Published:** 2019-07-12

**Authors:** Carl Jacob Holmberg, Gulan Alwan, Lars Ny, Roger Olofsson Bagge, Dimitrios Katsarelias

**Affiliations:** 1 Department of Surgery, Institute of Clinical Sciences, Sahlgrenska Academy at the University of Gothenburg, Gothenburg, Sweden; 2 Department of Surgery, Sahlgrenska University Hospital, Gothenburg, Region Västra Götaland Sweden; 3 Department of Oncology, Institute of Clinical Sciences, Sahlgrenska Academy at the University of Gothenburg, Gothenburg, Sweden; 4 Department of Oncology, Sahlgrenska University Hospital, Gothenburg, Region Västra Götaland Sweden; 5 Wallenberg Centre for Molecular and Translational Medicine, University of Gothenburg, Gothenburg, Sweden

**Keywords:** Melanoma, Abdominal metastasis, Metastasectomy, Tumor resection, M1c

## Abstract

**Background:**

Cutaneous melanoma has a rapidly increasing incidence in Sweden, and it has more than doubled in the last two decades. In recent years, new systemic treatments for patients with metastatic disease have increased overall survival. The role of surgery in the metastatic setting has been unclear, and no randomized data exist. Many surgeons still perform metastasectomies; however, the exact role probably has to be redefined. The aim of this single-institution study was to retrospectively examine the safety and efficacy of surgery in abdominal melanoma metastases and to identify prognostic and predictive factors.

**Methods:**

Retrospective analysis of a consecutive series of all patients with stage IV melanoma with gastrointestinal metastases that underwent abdominal surgery at a single center between January 2010 and December 2018. Fifteen patients who underwent in total 18 abdominal procedures, both acute and elective, were identified and included in the study.

**Results:**

Out of 18 laparotomies, six (33%) were emergency procedures due to ileus (*n* = 4), small bowel perforation (*n* = 1), and abdominal abscess (*n* = 1). Twelve procedures (66%) were elective with the most common indication being persistent anemia (58%, *n* = 7), abdominal pain and anemia (33%, *n* = 4), and abdominal pain (8%, *n* = 1). All procedures were performed by laparotomy. There were 19 small bowel resections, 3 partial colon resections, and 2 omental resections. Radical resection was possible in 56% (*n* = 10) of cases and 67% (*n* = 8) when only considering elective procedures. In 17 of 18 procedures (94%), there were mild or no surgical complications (Clavien-Dindo grades 0–I). The median overall survival was 14 months with a 5-year survival of 23%.

**Conclusions:**

Patients with abdominal melanoma metastases can safely undergo resection with a high grade of radical procedures when performed in the elective setting.

**Trial registration:**

ClinicalTrials.gov, NCT03879395. Registered 15 March 2019.

## Background

Skin melanoma has the highest increasing incidence of all malignancies in Sweden, and it has more than doubled in the last two decades. Incidence rates are rising globally as well, with annual increases as high as 4–6% in fair-skinned populations in the last decades [1]. In 2018, the reported age-standardized incidence in Sweden was 24.7 per 100,000, compared to 3.5 per 100,000 globally, giving Sweden the sixth highest incidence in the world [2]. Thicker melanomas (Breslow thickness > 4 mm), which have a significantly higher risk for metastasis and poorer prognosis, have more than quadrupled in the same time period. However, mortality figures have remained roughly unchanged, likely in part because of simultaneous major advances in systemic treatments [3].

Melanoma most commonly develops in the skin, but can also originate in the eye and in the mucosa of the gut, respiratory tract, and urogenital organs. For cutaneous melanoma, primary tumors are most often found on the lower extremities in women and on the trunk in men [3]. When metastases occur, melanoma can spread to any location and organ of the body and is staged according to the 8th edition of the American Joint Committee on Cancer (AJCC) TNM classification. Melanoma with distant metastasis is staged as M1, with subclasses M1a (distant skin, subcutaneous, or nodal metastases ), M1b (lung metastases), M1c (visceral metastases), and M1d (brain metastases) [4]. Metastases in the gastrointestinal tract are found in approximately 20% of stage IV patients, but previous autopsy studies have shown a prevalence as high as 58% in deceased patients. The most common sites of metastases are in the small bowel, followed by the large bowel and the stomach [5–7].

As previously reported, resection of abdominal visceral metastases can lead to a potential survival benefit and durable disease control [6, 7], and that it is feasible both in an elective [8] and in an acute setting [9]. The treatment of metastatic melanoma took a major leap forward with the introduction of immunotherapies using CTLA-4 and PD-1 antibodies as well as targeted therapies using BRAF/MEK inhibitors [10–13]. These treatments have opened up potentially new perspectives regarding the role of surgery for metastatic disease, and the role of surgery will have to be redefined.

The aim of this single-institution study was to retrospectively examine the safety and efficacy of surgery in abdominally metastatic (M1c) melanoma and to define possible prognostic and predictive factors, in order to identify stage IV melanoma patients that could benefit from surgery in the modern era of systemic therapies.

## Methods and patients

### Methods

We conducted a retrospective analysis of a consecutive series of all patients with stage IV melanoma with gastrointestinal metastases (M1c) that underwent abdominal surgery at a single institution between January 2010 and December 2018. The local database for registration and planning of surgeries was searched for patients with an ICD-10 code for melanoma combined with any code designating abdominal surgery. Both acute and elective surgeries were included. Pre- and postoperative data were gathered from our prospectively kept database and completed with data from the Swedish Cancer Registry and the Swedish Cause of Death Registry. Data were collected on patient demographics, timeline of diagnosis, primary tumor biology, staging, performance status, surgical interventions, surgical complications according to the Clavien-Dindo classification [14], pre- and postoperative systemic treatments, and survival. Tumors were staged according to the 8th edition of the AJCC staging system. Survival was defined as the time from surgery of abdominal metastasis to death or end of the study period (December 2018). Statistical analysis was conducted using SPSS (SPSS Inc., Chicago, IL, USA). Survival was calculated using the Kaplan-Meier method. All work is reported in line with the STROCSS criteria [15].

## Results

### Patient characteristics

During the 6-year period, a consecutive series of 15 patients underwent a total of 18 laparotomies (three patients underwent surgery at two separate occasions) including a total of 30 different surgical procedures (Table 1). The majority of the patients were male (80%, *n* = 12). The median age at diagnosis of the primary melanoma was 65 years (range 28–75), and the most common site of the primary melanoma was the torso (40%, *n* = 6). The median time from diagnosis of the primary melanoma to metastases was 45 months (range 0–173) and a median time of 3.6 months (range 0.5–46.9) until surgery. The median age at the time of surgery was 69.1 years (range 35.4–85.7), and 67% (*n* = 12) of the patients received preoperative systemic treatment. A BRAF-V600E/K mutation was identified in 47% (*n* = 7) of the patients.

**Table 1:** Collected patient data

No.	Gender	Age	TNM at surgery	Primary site	BRAF status	Time diagnosis to M1c	Pre-op treatment	Time M1c to surgery	Surgery	Radicality	Indication	Elective/emergency	WHO performance status	Complication (Clavien-Dindo), type	Length of stay (days)	Stay at ICU (days)	Post-op treatment	Status	Survival from surgery	Survival from M1c
1	Male	63	T0N3M1c	Unknown	V600E/K	25	C, I, R (lumbar spine)	4.2	Enterectomy + fistula and abscess	R0	Sepsis, abscess	Emergency	1	II, antibiotics postop	11	0	R (brain)	DWD	3.9	8.1
2	Male	73	T3bN0M1c	Back	wt	40	0	2.8	Enterectomy, part. colectomy	R0	Bleeding, anemia	Elective	1	0	8	0	R (brain)	DWD	17.1	19.9
3	Female	51	T2aN0M1c	Left leg	V600E/K	94	0	0.5	Enterectomy	R2	Pain, bleeding	Elective	1	0	7	0	C, I	DWD	14.9	15.4
4	Male	72	T3bN1aM1c(0)	Back	wt	50	C	4.1	Enterectomy x 2	R2	Pain, anemia	Elective	1	I, long time to oral intake	8	0	C	AWD	58.6	62.7
4 (2nd)	*	*	T3bN1aM1c(0)	*	wt	50	C	19.1	Enterectomy, appendectomy	R0	Pain, anemia	Elective	0	0	4	0	I	ANED	43.6	62.7
5	Male	39	T4aN0M1c(0)	Left arm	wt	23	0	0.8	Enterectomy	R0	Pain, anemia	Elective	1	0	5	0	0	ANED	55.4	56.2
6	Male	71	T1bN0M1c	Back	wt	139	0	1.6	Enterectomy x 2	R0	Anemia	Elective	1	I, bradycardia	8	0	0	DWD	43.3	44.9
6 (2nd)	*	*	T1bN0M1c	*	wt	173	0	36.8	Part. colectomy + omentum	R0	Anemia	Elective	2	IIIb, wound rupture and reoperation	16	0	0	DWD	8.1	44.9
7	Male	68	T0N3bM1d(0)	Unknown	wt	4	C	4.6	Enterectomy	R2	Ileus, pain, anemia	Emergency	1	0	9	0	C	DWD	12.8	17.5
8	Male	75	T0N2cM1c(1)	Unknown	wt	0	C	1.3	Enterectomy x 2	R2	Small bowel perforation	Emergency	2	II, antibiotics postop	25	1	0	DWD	1.0	2.3
9	Female	28	T1aN1aM1c(1)	Back	V600E/K	52	C, I	34.7	Debulking of small bowel mesentery	R2	Ileus, pain	Emergency	2	I, PCA pump	15	0	C, I	DWD	18.5	53.3
9 (2nd)	*	*	T1bN1aM1c(1)	*	V600E/K	52	C, I	46.9	Enterectomy x 2, debulking	R2	Pain, anemia	Emergency	2	I	14	0	C, I	DWD	6.3	53.3
10	Male	52	T0N2bM1c(1)	Unknown	wt	0	C, I, R (axilla)	8.0	Part. colectomy + abdominal wall	R1	Pain	Elective	1	I	8	0	C, I	DWD	13.9	21.9
11	Male	69	T4bN1aM1c(1)	Back	V600E/K	36	0	1.5	Enterectomy x 2	R2	Anemia, bleeding	Elective	3	I	21	0	I	DWD	3.8	5.3
12	Male	52	T4bN2M1c(0)	Right temple	wt	3	C, I	37.7	Enterectomy	R0	Anemia	Elective	0	0	6	0	I	ANED	16.7	54.5
13	Female	58	T3aN3bM1c(1)	Right arm	V600E/K	63	C, ILP, I	46.0	Enterectomy	R0	Ileus, pain, anemia, bleeding	Emergency	3	0	13	0	C	DWD	13.7	59.7
14	Male	63	T4bN0M1c(0)	Right leg	wt	84	C, I, R (lung)	0.8	Enterectomy	R0	Anemia, bleeding	Elective	1	I, gastric retention	7	0	C, I	AWD	11.3	12.1
15	Male	67	T1aN2bM1c(0)	Thorax	V600E/K	14	R (axilla)	3.1	Enterectomy + omentum	R0	Anemia, bleeding	Elective	1	II, blood transfusion	5	0	0	ANED	2.9	6.0

Time measured in months unless stated otherwise

*2nd* patients’ second surgery, *WT* wild type, *R* radiotherapy, *C* chemotherapy, *I* immunotherapy, *ILP* isolated limb perfusion, *DWD* dead with disease, *AWD* alive with disease, *ANED* alive, no evidence of disease

*Same as above. Indicating that the patient/information is the same as the one above it

### Indications

Out of the 18 laparotomies, 33.3% (*n* = 6) were emergency procedures and 66.6% (*n* = 12) were elective procedures. The most common primary indication for emergency surgery was ileus (*n* = 4), followed by small bowel perforation (*n* = 1) and abdominal abscess (*n* = 1). The most common primary indication for elective surgery was persistent anemia (58%, *n* = 7), followed by abdominal pain and anemia (33%, *n* = 4) and abdominal pain (8%, *n* = 1). The most common symptoms overall were persistent anemia (72%, *n* = 13), abdominal pain (50%, *n* = 9), and acute rectal bleeding (33%, *n* = 6). The three patients that underwent surgery at two separate occasions did so because of recurrence of symptomatic intraabdominal tumors.

### Type of surgery

**Fig. 1:**
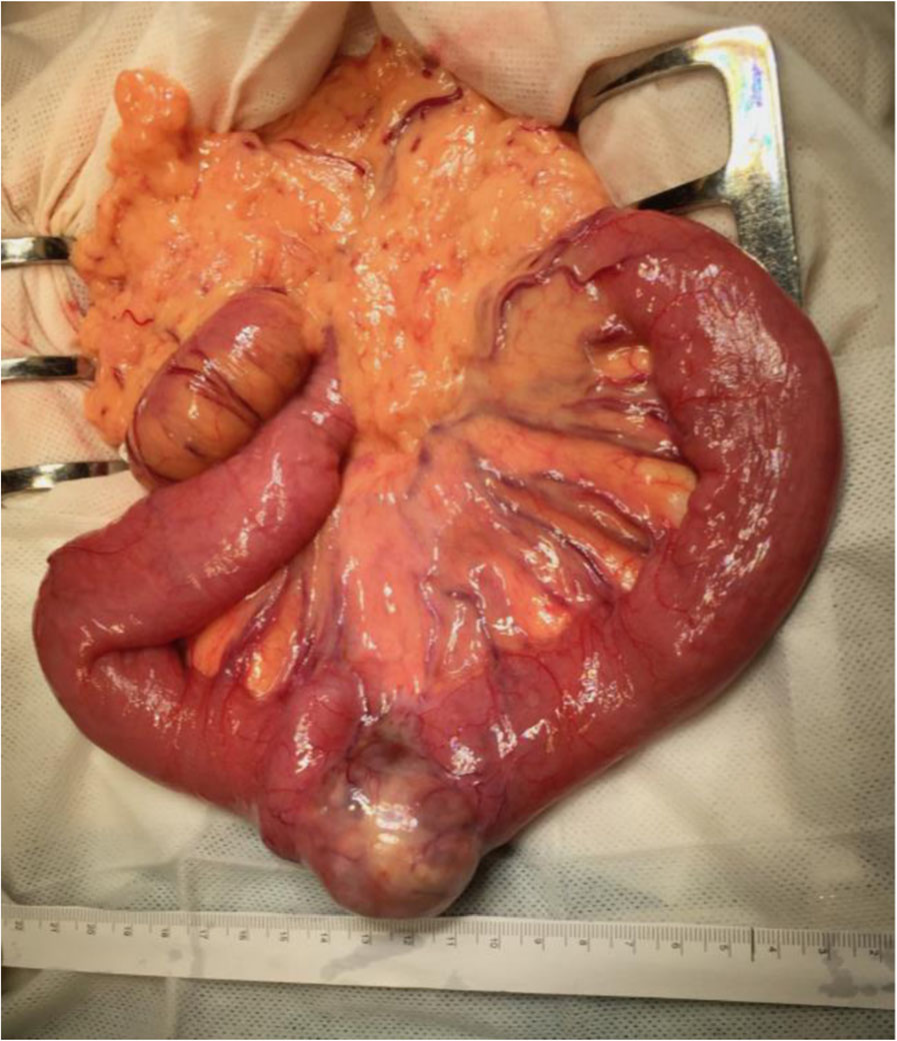
Perioperative photo of small bowel melanoma metastasis

All 18 operations were performed as open laparotomies, and there were a total of 30 separate intraabdominal procedures. The most common surgical procedures were small bowel resection (Fig. 1) (*n* = 19), followed by partial colectomy (*n* = 3) and omental resection (*n* = 2). Primary anastomosis was feasible in 20 of the 22 enterectomies (91%). Seven patients underwent more than one resection, and more than one bowel anastomoses were performed at the same time. The operation was considered radical (R0) in 56% (*n* = 10) of the procedures, as stated by both macroscopically evident intraabdominal tumor removal and microscopically free margins. In 70% (*n* = 7) of those R0 operations, the resected tumors were the only known metastases and surgery was thereby performed with the intention to achieve a disease-free status. In one patient, the resection was deemed macroscopically radical but re-categorized as non-radical after histopathological analysis showed tumor-positive margins (R1). In the remaining cases (39%, *n* = 7), tumor infiltration was too extensive to allow for anything but debulking (R2).

### Complications

The median length of postoperative stay was 8.0 days (range 4–25). Only one patient was admitted to the intensive care unit for a duration of 1 day. This was also the only patient who died in the immediate postoperative period, due to precarious conditions before surgery which was performed acutely, and this death was therefore not regarded as a surgery-related complication. The majority of procedures (88%, *n* = 16) resulted in no or mild surgical complications (Clavien-Dindo grades 0, I, or II), and only one case resulted in a complication requiring re-operation (Clavien-Dindo grade III) because of wound dehiscence.

### Survival

**Fig. 2:**
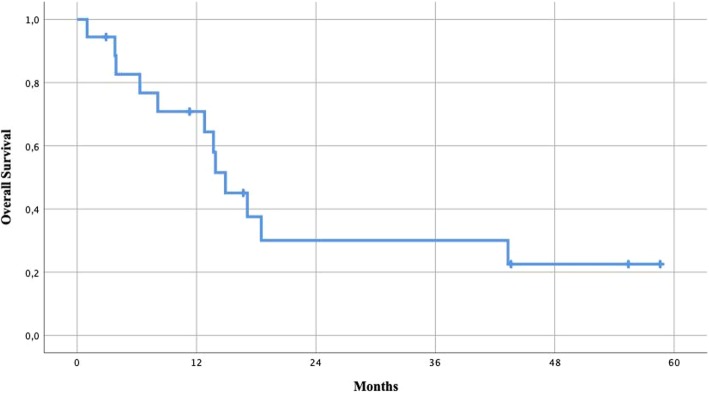
Overall survival after abdominal surgery

Of the 15 patients included, ten died of recurrent disease, one is alive with recurrent disease, and four are alive with no evidence of disease. The median overall survival was 13.8 months (range 1.0–58.6), and the 5-year survival was 22.5% (Fig. 2). The median survival from diagnosis of M1c-status was 33.4 months (range 2.3–62.7).

**Fig. 3:**
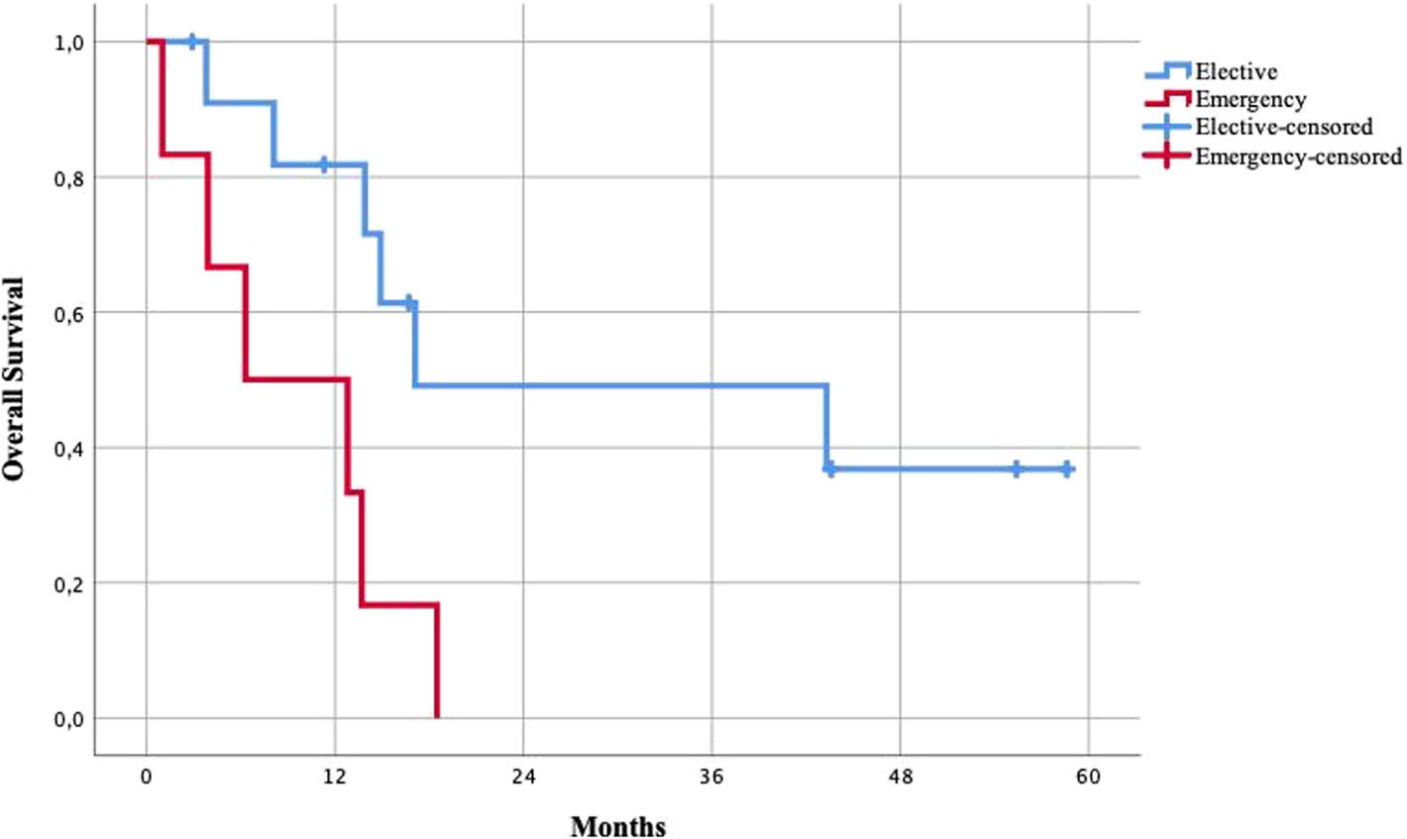
Survival of patients undergoing elective vs emergency surgery

**Fig. 4:**
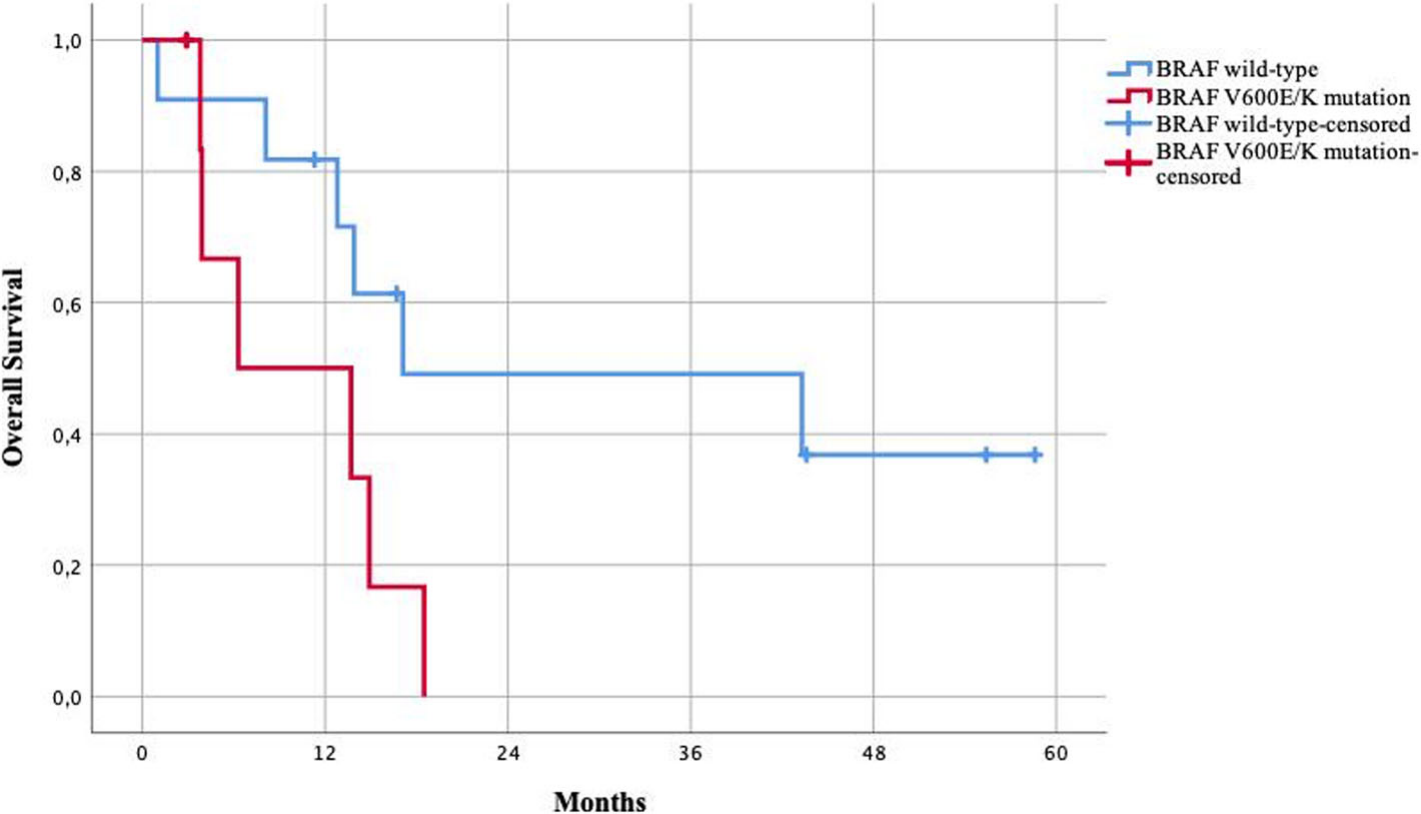
Survival of patients with BRAF wild-type vs. V600E/K mutation

**Fig. 5:**
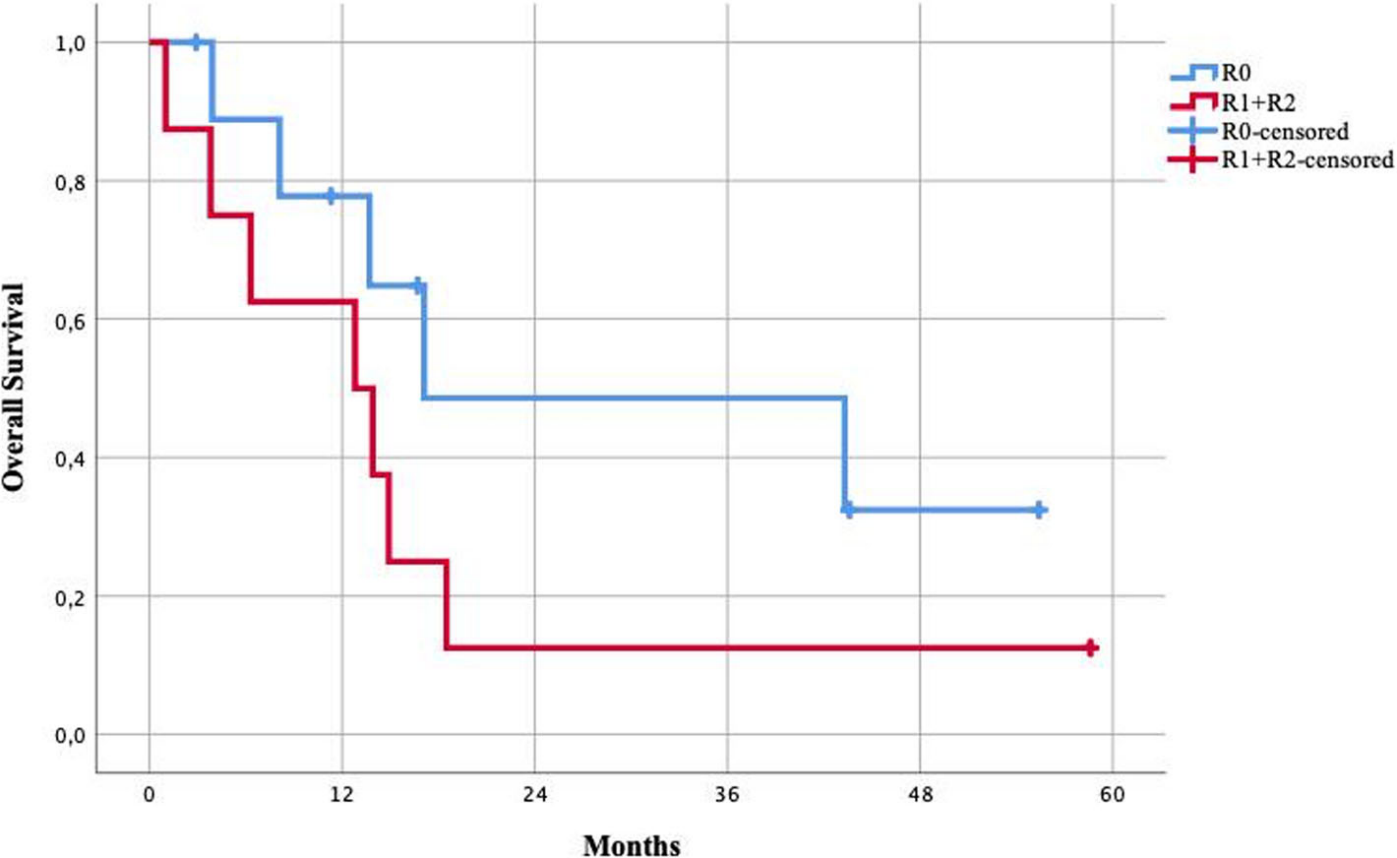
Survival of patients by radicality, R0 vs. R1+R2. R0, radical resection; R1, macroscopically clear but microscopically non-radical; R2, macroscopically non-radical tumor debulking

Elective surgery, absence of BRAF wt (wild type), and radical resections were all associated with longer survival. Patients undergoing emergency procedures had lower survival rates compared to patients undergoing elective procedures, with a 1-year survival of 82% vs 50% and a median survival of 16 months vs 10 months (Fig. 3). Patients with a BRAF V600E/K mutation (*n* = 6) had significantly lower survival compared to the wild-type phenotype, with 1-year survival of 50% vs 82% and median survival of 6 months vs 17 months (Fig. 4). Patients undergoing surgery with radical resections (R0 and R1) had higher survival rates compared to patients undergoing debulking of metastasis (R2), with 1-year survival of 78% vs 57% and a median survival of 15 vs 13 months (Fig. 5).

## Discussion

In this exploratory single institutional retrospective case series, we have shown that patients with abdominal metastases of melanoma can safely undergo metastasectomy in both an emergency and an elective setting. By resection of intraabdominal tumors, symptoms could be alleviated and oncological treatment could in many cases be continued, thus offering patients an adequate palliative procedure with higher chance of prolonged survival.

There were no significant logistical delays for patients necessitating surgery, and as such, the median time from M1c-status to surgery is accurately reflecting the time from metastatic disease symptoms to surgical intervention. Most patients were in an overall good health status at the time of surgery, with two thirds (*n* = 12) having a WHO Performance Status of 0–1.

BRAF mutation was associated with lower survival, which is in accordance with previously reported data from larger studies [16]. However, in this series, there was a significant overlap in the groups with BRAF wt tumors and elective procedures with nine out of 12 patients undergoing elective surgery being BRAF wt, which may explain the difference in survival. Patients with BRAF wt tumors, undergoing elective surgery with radical resection of the metastasis, generally had the best outcome in this study. In view of these findings, BRAF wt, elective surgery, and radical resection were identified as prognostic factors for survival; however, the study is small, and thus, no definitive conclusions can be drawn.

The median overall survival from surgery in our material (13.8 months) is lower than, e.g., what Sosman et al. [17] found in their SWOG-trial (21 months). Possible explanations are that the patients in our study were generally in poorer condition with a worse performance status and higher median age at the time of surgery, that a significant part of our surgeries was performed acutely as opposed to only electively, and that we had a lower frequency of complete tumor resections. Furthermore, systemic therapy regimes are also likely to vary greatly between the two studies, confounding any direct comparison. Our finding that the most common symptoms in this patient group are anemia, bleeding, and abdominal pain is in line with what has been previously reported by, e.g., Ollila et al. [6].

This study has several limitations, foremost the small sample size. As such, although surgery of abdominal metastasis has been deemed safe in this cohort, it is not possible to generalize this conclusion to the all stage IV melanoma patients. Furthermore, we have limited the scope of the study to resection of symptomatic metastases, and no conclusions can be drawn regarding the resection of asymptomatic tumors. Patients in this study underwent surgery mainly to reduce symptoms, but secondary also in order to allow for continued oncological treatment. A possible survival benefit of metastatic surgery alone is not possible to infer in the absence of a control group.

The future role of melanoma metastasectomy in an era of effective systemic treatments is not yet clear. Palliative symptomatic tumor resections will continue to be of value in advanced disease. The use of surgery in patients with isolated or oligometastatic disease that do not respond to systemic treatments will likely increase in the coming years and needs to be explored in a systematic fashion. Also, as the personalized targeted cancer treatments seen in the last decade continue to develop, the need for surgical biopsies of tumor tissues for analysis will develop with it [18, 19].

Further studies are needed to better understand the role of metastatic surgery in treating abdominal stage IV malignant melanoma. Randomized trials investigating the combination of surgery and modern effective systemic treatments would be required to fully establish the role of surgery in the future, but that requires the surgical oncology community to develop such protocols in tight collaboration with medical oncologists.

## Conclusions

Patients with abdominal melanoma metastases can safely undergo resection with a high grade of radical procedures when performed in the elective setting. Further studies are needed to better guide clinical surgical decisions in patients with abdominal melanoma metastases.

## Data Availability

All data generated or analyzed during this study are included in this published article.

## Abbreviations

AJCC: American Joint Committee on Cancer
wt: Wild type
